# Supplementary material for: Hyperendemicity of cysticercosis in Madagascar: Novel insights from school children population-based antigen prevalence study
Source: PLoS One. 2021 Oct 1;16(10):e0258035. doi: 10.1371/journal.pone.0258035 (PMC8486106; doi:10.1371/journal.pone.0258035)
Supplement: Attachment — Submitted filename: PONE-D-20-27048 Response to reviewers V2.docx [file pone.0258035.s001.docx]

PLOS ONE

Hyperendemicity of Cysticercosis in Madagascar: Novel insights from a school population-based antigen prevalence study

--Manuscript Draft--

| Manuscript Number: | PONE-D-20-27048 |
| --- | --- |
| Article Type: | Research Article |

Answers to the Reviewers.

Reviewer #1: This manuscript describes a serological survey of school children in 7 major cities of Madagascar for the presence of circulating antigen being indicative of infection with viable cysticerci of *Taenia solium*. They found that up to 37% of the children were positive, with an average prevalence of 27%. The authors point out that this is the highest prevalence described for anywhere in the world, after one village in DRC where a seroprevalence of 21% was recorded.

The data in the paper give no confidence that the serological results are valid. The claimed level of active infection is either a disaster of extraordinary proportions for the school children of Madagascar, or an indication that the serology was faulty. We are provided with no data whatsoever about how or where the serology was performed. Two references are given as the serological methodology, published by the last author. Was the serology performed in in Madagascar or in Belgium in the senior author’s laboratory? How can we know the performance characteristics of the assay outside the senior author’s laboratory if the tests were undertaken in Madagascar? What control samples were tested from known cases of cysticercosis and, more importantly, from persons who have no history of cysticercosis? Did the authors source any negative controls (at least persons with no history of symptoms suggestive of being infected with *T. solium*) from the Malagasy population, such as members of the project team?

In the complete absence of any information to support the claimed extraordinarily high prevalence of cysticercosis in Malagasy children, and the complete absence of any information about the negative human controls used when the tests were performed, or where the tests were undertaken, the paper conclusions cannot be regarded as robust.

We acknowledge that the Ag-ELISA used in the current study is an in-house test and therefore, we understand the concerns raised by reviewer #1 on the validity of the results. All tests were done in the laboratory of Pasteur Institute, Madagascar using reagents that were provided by the Institute of Tropical Medicine (ITM), including the two monoclonal antibodies that are produced at ITM. Before the start of the study, laboratory technicians of Pasteur Institute received a two-weeks training on the test in Madagascar by an ITM technician. Standard operating procedures were provided to the Pasteur lab and the results of each plate were sent to ITM for quality control. The procedure involves the use on each ELISA plate of a substrate control, a conjugate control, two positive control serum samples and a set of 8 negative control serum samples; the optical densities of the latter being used for the calculation of the cut-off as described in the manuscript. Both the positive and negative controls were provided by ITM. The two positive controls are diluted serum samples from highly infected individuals. They are used as a quality check on the plates. The 8 negative controls originate from non-infected individuals of Belgian origin, and are the same samples than those used at ITM and consequently served as an extra quality check of each plate. In contrast to antibody tests that may show a background as a result of local conditions such as exposure to other pathogens, no such a background has been observed in the Ag-ELISA following the routine TCA pre- treatment of the serum samples before application in the ELISA.We therefore believe that our laboratory results are robust enough to make the conclusions as described in the paper.

We have made changes accordingly in the Materials and Methods section.

Reviewer #2: The manuscript needs a MAJOR revision.

Major comments:

1. Abstract: Should make clear that the samples were from 13 years ago (assuming that is the case), so the conclusion might not be valid, unless they speculate that risk factors haven’t changed.

The sera were collected in 2007. They were stored at 2-8°C for a maximum of 48h then sent to Pasteur Institute of Madagascar where they were kept frozen at -20°C.The *T. solium* Antigen analysis were performed in 2008. According to Word Bank data (accessed 12/21/2020): GDP per capita (current USD) decreased from 536 to 523 USD (<https://data.worldbank.org/indicator/NY.GDP.PCAP.CD?locations=MG>). Assuming that the risk factors are associated with poverty and haven’t significally changed, the obtained data may be extrapolated to the current years.

We have made changes accordingly in the conclusions of the discussion section.

2. Study design is not clear, it is only a geographical description.

This is a cross-sectional studyin schoolchildren from seven sites in the four different biotopes of Madagascar.This research was not an ecological study but a cross-sectional study. Therefore, this study was not based on aggregated data and individual data were directly analyzed. The title has been changed in order to be more specific “school children, antigen prevalence study”.

3. Timelines are not clear through the manuscript.

When were the samples collected? Between February and March 2007 When were the tests performed? Tests have been performed in 2008.

4. Analytical procedures:

4a. Which methods were used to develop the ELISA? The Ag-ELISA hasn’t been developed but implemented at the IPM. It was developed at the ITM, Antwerp. The reagents were provided by the ITM(see response to comments of reviewer#1).

4b. Where were the reagents produced? at the ITM, Antwerp(see response to comments of reviewer#1).

4c. Source of positive controls? ITM, Antwerp (see response to comments of reviewer#1).

4d. Source of negative sera? Are they from the same population? ITM, Antwerp (see response to comments of reviewer#1).

4e. Any positives confirmed as positives? Since no gold standard is available for the diagnosis of Neurocysticercosis, positivity is assessed by clinical, imaging and pathological or biological data (Del Brutto criterions).

5. Statistical analyses:

5a. The analysis by age is not clear. Which groups were analysed? What are the results by group?

This information is not clearly enough in the paper. The age was collected as a continuous variable, and was then treated as a dichotomous variable. We used the population mean as a cut-off and chose the "under 8" category as the reference variable during the analysis. This analysis was also conducted according to the age status. The results are presented in a supplementary table.

| Characteristic | Categories | Total | | Ag ^+^ | | Ag ^-^ | |  |
| --- | --- | --- | --- | --- | --- | --- | --- | --- |
|  |  | n | % | n | % | n | % | P-Value |
| Sex | Male | 887 | 50.83 | 250 | 51.76 | 637 | 50.48 | 0.63 |
|  | Female | 858 | 49.17 | 233 | 48.24 | 625 | 49.52 |  |
| Age (years) | < 8 | 799 | 45.79 | 191 | 39.54 | 608 | 48.18 | <0.001 |
|  | ≥ 8 | 946 | 54.21 | 292 | 60.46 | 654 | 51.82 |  |

5b. Why try to relate prevalence to altitude and rainfall, and not to the well-known and necessary risk factors such as presence of backyard pigs and open defecation?

As mentioned in the Materials and Methods section the blood samples were collected in the framework of a malaria study. Therefore, factors recorded are not specific to cysticercosis. Moreover, it is well known that open defecation and free roaming of pigs are factors facilitating the transmission of *T. solium.* In this approach, the objective was to explore the potential influence of environmental characteristics ,altitude, rainfall, bioclimatic patterns. This point is rarely explored.

6. Results: Results by age are not clear.

Please, refer to 5a. answer.

7. Conclusion:

7a. If the serum is indeed from 2007, 13 years later, many epileptic cases would have been detected. Is this the case? What is the rate of epilepsy? Before the study, all the sera were anonymized, it was not possible to carry out a follow-up. The protocol has been validated by the national ethics committee of Madagascar. The aim being to help the government on the basis of grouped data to take appropriate action at the local, regional or national level. No data regarding this study are therefore available for assessing the epilepsy rate. Furthermore, in some cases blood positivity may not be related to a neurological localization.

8. Ethics: need to justify why the children were not followed-up as NCC is potentially very dangerous (I assume it is because of timings, but as they are not specified, it is not clear). Before the study, all the sera were anonymized, it was not possible to carry out a follow-up. The protocol has been validated by the national ethics committee of Madagascar. The aim being to help the government on the basis of grouped data to take appropriate action at the local, regional or national level.

Minor comments:

9. Line 54: Use original reference for 30% seizure disorders (Ndimubanzi et al, 2010). We thank you for your input, the modification has been made.

10. Line 56: Andean area of South America is not correct (Argentina is not endemic for example). Better change to areas of South America. We thank you for your input, the modification has been made.

11. Figure 1: There is no legend to interpret the colours. The dark blue is difficult to read. What does it mean 237 in the title? We thank you for your input, 237 mention was an error, the modifications have been made.

12. Table 1: Only last row is Ag prevalence. Title is not appropriate. Delete % sign from first results column. We thank you for your input, the modification has been made and the title has been modified accordingly.

13. Editing – in several places spaces before brackets are missing. We thank you for your input, the modification has been made.

Reviewer #3: This is a very interesting manuscript showing that cysticercosis is infecting children at a very high prevalence in urban areas of Madagascar. The authors should however consider the following to improve it

1) the authors should follow the STROBE guidelines for reporting observational studies in epidemiology.The STROBE guideline for cross-sectional studies has been followed and as for this new revised manuscript, modifications have been done accordingly

2) the sampling frame and study sites are not clearly described; there is a mismatch between the text and Fig1, and Fig1 title is unclear. How many sites in the highlands? Why more than 2 sites in the other areas? In each biotope two sites (=town) were selected except for highlands (=1 town) for logistical reasons. We took your input into account: the figure has been improved, all seven study sites have been included. The title has been clarified.

3) given the high prevalence of infection, a log-binomial model would be more appropriate here and make interpretation of ratios more straightforward. As you advised us, we carried out the analysis from a log-binomial model. The results of that model were similar to those obtained with the multilevel logistic regression model.

It is also unclear if the biotype was included as a level in the hierarchical model or as a variable (only 2 levels are mentioned in the description). Each level used in the cluster sampling should be included in the multi-level model. it is unclear if this was done or not. Thank you for that comment. As described in Table II, the variable "Biotype" consists of four modalities. The "Highlands" biotype is the reference modality for the hierarchical model. This variable was introduced as a fixed effect and did not define a hierarchical level (in part because of the very low number of different level units (one of the conditions to perform such models is a minimum of units of each level). Only two levels were available and were introduced in the multilevel model ; level one : child and level two : city. The school level was not entered in the database. The following text was inserted in the manuscript, line 137, page 14 : « Sex, age, biotope, altitude, temperature and rainfall were introducted in the models as fixed effects. »

4) More details on sampling of households and children within each town (is town=site?) are needed. At this point, it is unclear if towns were randomly selected and how children within town were selected. This could introduce selection bias. A two-level cluster random sampling (school and classroom), including schoolchildren from either public or private school was used to select the study population. Depending on the site (=town),samples were collected from one (in Tsiroanomandidy and Miandrivazo), two (Andapa, Ejeda and Moramanga) or three schools (Farafangana, Ihosy and Maevatanana). Clarifications have been added to the text line 87-100).

5) It seems very odd for a child aged 3 years old to be considered as school aged. Do 3-year olds go to school in Madagascar? Yes, this data has been confirmed.

6) Denominators and 95%CI should be reported in Table 1 for ease of reading and interpretation. Thank you for this remark. Rainfall, altitude and temperature are contextual variables, i.e. the same value is affecting all the subjects of the same geographical site, so no variability is considered inside each site. The 95% confidence interval around the proportion of Ag+ and the number of missing data was added per site in Table2.

7) Why was the age dichotomized and was effect modification evaluated? As major epidemiological factors, age and sex were introduced in the analysis. These classical variables were retained to ensure the absence of confusion bias. This could indeed be feared for age (significantly linked to seropositive status).The following text was inserted in part of the results section : « As major epidemiological factors, age and sex were introduced in the analysis. »

The used of dichotomized age allowed us to test for different effects, such as interaction and to more easily cope with a potential problem of linearity assumption.

The following text was inserted in the statistical part of the material and methods section :  « Interaction between variables in models were tested. None of them were significant or improved the models. » (143-144)

Were there any other confounders assessed? SES for one could be an important confounder of the impact of even the environmental factors. General sanitation even at the site level is also important to adjust for, it may be confounding the association with environmental factors. What about ethnicity?

A supplementary table showing the distribution of ethnicities is presented here. The dispersion of the population was very important, unbalanced and concerned 20 ethnic groups. We considered it relevant not to include it in our model. The following text was inserted in the results part : « Twenty ethnic groups are represented in the study population. Most of the children where of Merina or Betsileo ethnic group.” » (152-154).

| Ethnies (N= 1744) | Number | Pourcentage |
| --- | --- | --- |
| Merina | 507 | 29,07% |
| Betsileo | 417 | 23,91% |
| Betsimisaraka | 40 | 2,29% |
| Sakalava | 45 | 2,58% |
| Antaisaka | 137 | 7,86% |
| Antandroy | 76 | 4,36% |
| Mahafaly | 76 | 4,36% |
| Vezo | 10 | 0,57% |
| Bara | 49 | 2,81% |
| Antakarana | 5 | 0,29% |
| Antemoro | 103 | 5,91% |
| Antaifasy | 79 | 4,53% |
| Masikoro | 0 | 0,00% |
| Antambohoaka | 1 | 0,06% |
| Tsimihety | 95 | 5,45% |
| Tanala | 9 | 0,52% |
| Bezanozano | 2 | 0,11% |
| Sihanaka | 5 | 0,29% |
| Antanosy | 11 | 0,63% |
| Zafimaniry | 4 | 0,23% |
| Foreigners | 73 | 4,09% |

There needs to be a Table of univariate analyses. Following the STROBE guidelines would help in this regard.

Thank you for that remark. We have added a complementary table describing the explained variables according to sex and age status. Altitude, rainfall, temperature and biotope are contextual variables. Univariable analyses are presented in Table II.

8) a more solid discussion of the impact of the environmental factors is needed with strenghts and limitations

Few data are available on the impact of the environmental factors, such as rain, altitude and temperature, toward parasites survival in the environment. However as quoted, sociological and behavioral practices added to the sanitation amenities may also be related to a specific environment and may play an even bigger role in the process of breaking or enhancing the parasitic cycle.

9) There needs to a paragraph on limitations

This section has been emphasized in the discussion section.

Specific comments

Line 60-61: please provide a range of prevalence found with Ag ELISA in SSA (like what is done for Ab). There is also a relatively recent systematic review of prev of HCC in SSA which could be used here (Coral Almeida 2015),Thank you for your input, it has been taken into account.

Could there be more than 1 child per household? If so, was this considered in the analyses?

Yes, most households do have more than 1 child but this data was not available and couldn’t be handled in our model.

Study design: the DATES of the survey must be reported. The dates are Between February and April 2007, they have been reported.

Table 2 and related analyses: re-run the analyses with the Equatorial region as a reference for easier interpretation.

As requested, Table II has been adapted and presents the equatorial region as a reference. It is now Table III in the new version.

Lines 175-176: this is not quite correct. The EITB performs a lot better in this situation, though not perfect. Also, the AgELISA also performs poorly when there is only one NCC cyst, so this argument is incorrect the way it is stated here.Thank you for these relevant remarks. Some modifications have been applied to the text (189-190).

Lines 206-209: the big difference here is that most other studies were conducted in adults where the effect gender is often modified by age and is an indirect measure of behavior. This should be noted here.Thank you for your input, it has been taken into consideration and the conclusion has been modified accordingly (208-210).

To the Editor:

If figure 1 is original, then there is no copyright issue.  Can you please provide details within your Response to Reviewers document to confirm that the figure used is original.

Madagascar's layer was drawn using geodata from World of Maps ( [www.worldofmaps.net](http://www.worldofmaps.net)), and mapping operations were performed using QGIS 2.18 software. Source: QGIS Development Team. QGIS Geographic Information System. Open Source Geospatial Foundation.; 2009. Therefore, this map is not copyrighted
