# Supplementary material for: Hyperendemicity of cysticercosis in Madagascar: Novel insights from school children population-based antigen prevalence study
Source: PLoS One. 2021 Oct 1;16(10):e0258035. doi: 10.1371/journal.pone.0258035 (PMC8486106; doi:10.1371/journal.pone.0258035)
Supplement: Attachment — Submitted filename: Response to reviewers Final (V3).docx [file pone.0258035.s002.docx]

PLOS ONE

Hyperendemicity of Cysticercosis in Madagascar: Novel insights from school population-based seroprevalence study

--Manuscript Draft--

| Manuscript Number: | PONE-D-20-27048 (V3) |
| --- | --- |
| Article Type: | Research Article |

Answers to the Editor and to Reviewer(s)

*Editor: I agree with reviewer # 5  the authors need to discuss the limitations of the study and critically discuss the obtained data. The main limitation is the lack of clinical data and risk factors associated with cysticercosis. This concern was raised in the first peer review and needs to be properly discussed as suggested in the second revision. The authors should also address the possibility of the transient Ts antigen detection and discuss it accordingly.*

*Reviewer #5:  The paper reports the results of a study carried out in Madagascar to evaluate the prevalence of Taenia solium circulating antigen (Ts-antigen) in school age-children of seven major cities. The presence of Ts-antigen is considered indicative of infection with viable cysticerci of Taenia solium, although it may be a transient phenomenon with possible sero-reversion, hence not necessary established infection. The prevalence of Ts-Ag was 27%, with percentage up to 37% in some children groups. The observed Ts-Ag prevalence rate exceeds all antigen prevalence rates described worldwide, after one village in DRC where a seroprevalence of 21% was recorded.
The obtained results are of some interest but should be presented evidencing the limitations of the study and critically reviewing obtained data.*

*The study was conducted on serum samples collected in a malaria survey in 2007. Information on risk factors for cysticercosis (e.g presence of backyard pigs and open defecation) was not collected. No clinical data related to cysticercosis (epilepsia) are available neither for tested subjects nor local population. No data are available about teniosis. The authors explored the potential influence of environmental characteristics, altitude, rainfall and bioclimatic patterns.

The first question/doubt could be about the performance of the test, mainly its specificity, but also about the correct procedures of an in-house test. The authors affirm that tests were done by well-trained laboratory technicians of Pasteur Institute (Madagascar) by using reagents provided by the Institute of Tropical Medicine of Antwerp, where the test was developed and validated. Negative and positive controls were included and the procedures were correctly followed. The results of each plate were sent to ITM for quality control. Based on this, results seem to be robust.


The main limitations of the study are the lack of information about risk factors for cysticercosis and clinical data (mainly epilepsia) at the moment of enrollment (2007) and in subsequent years, as well as data on teniosis. It would be interesting to address this gap through other research activities in the studied areas. If data were already available, although preliminary, they could be mentioned in the paper.*

*Moreover, it is known that the presence of cysticercal circulating antigen may be short-live and sero-reversion is a common phenomenon. The authors mention this point in the final part of the discussion citing the paper of Mwape et al (ref 37). I suggest to read carefully the paper of Mwape et al reporting the results of a longitudinal study in Zambia with blood sampled three times, with a 6-month interval and analyzed for the presence of cysticercal circulating antigens and specific antibodies. Mwape et al paper should inspire the authors to present their results more critically and to revise the discussion.*

We thank you for your input. Although specific risk factors for cysticercosis were not available in this study, other relevant environmental factors were discussed as possible risk factors. The main benefits of this study are that

- we benefited from an extensive amount of multi-centered sera collected in 2007 amongst whole schoolchildren communities
- we used the B158/B60Ag-ELISA, a diagnostic tool for cysticercosis that has proven to produce valuable results in many studies but that has never been used in Madagascar.

As stated by both Editor and reviewer #5, we acknowledge the lack of both clinical and specific risk factors. These could have enabled us to better understand our results. As suggested, we rewrote our discussion, strengthening on the limitations of our study and trying to link our results with available information on epilepsy in Malagasy children.

We have made changes accordingly in the discussion section, as follows (in red in the text):

## Discussion

The seroprevalence of cysticercosis based on antibody detection ranked Madagascar within the highly endemic countries. The lack of sensitivity of Ab-ELISA’s may have underestimated the prevalence rates published in Madagascar. Most NCC cases (the most common clinical form of cysticercosis) found on the island are related to single cyst carriers, which are known to be difficult to detect with both antibody and antigen-based serological methods (26,27).

In contrast to the presence of Ts Ab, indicating exposure to the infection, the Ts Ag rate indicates the presence of active cysticercosis. With an average of 27% positives in children in our study, the Ts Ag prevalence rate exceeds all antigen prevalence rates described worldwide. High Ts Ag rates indicates that active infections with *T. solium* metacestodes occur within the Malagasy population, affecting the younger age class and that it is widespread on the island even though a visible heterogeneity was observed between the sites. Recent studies have shown that in African countries higher apparent prevalence of active infections occurred than in Asian countries (28). This difference may reflect epidemiological differences, especially a higher exposure; however, variations in host susceptibility and in parasite genotypes may also play a role (29,30).

Previous evidence based on antibody detection has indicated that all provinces of Madagascar were endemic for cysticercosis with significant differences between the areas. The use of the Ts Ag ELISA showed the same trends with a lower Ts Ag prevalence in the equatorial biotope. This supports the hypothesis that spatial heterogeneity in the distribution of infections may be influenced by environmental conditions, highlighting the interplay between socio-economic, behavioural and environmental factors in *Taenia*(31). Selection bias may also be taken into account. One major difference of our study is that most other studies were conducted in adults in which the effect of gender is often modified by age and is an indirect measure of behaviour. Biotopes are represented by solely one or two sites with sometimes marked variations in environmental or cultural behaviours. The access to fresh water is enhanced in the equatorial area with better hygiene practices, and less common pig raising and pork consumption than in the highlands (32). These conditions and practices may play a role in limiting the disease’s prevalence. While arid areas should conversely experience a lower survival of parasitic eggs (33), in the present study these areas were associated with high Ts Ag prevalence rates, just below the highlands’ rate. When considering the effect of desiccation on *Taenia* egg survival (33), this high Ag prevalence was not expected and requires further study.

An effect of gender on prevalence was not observed (20), unlike in studies in Latin America and other African countries. This might be due to cultural differences between populations(28). However, age was reported as a significant risk factor in the bivariate model for the presence of circulating antigens as quoted in different reports (28,34,35). Worldwide, one third of the total epilepsy cases arise in childhood and neurocysticercosis is a major cause, particularly in developing countries (35,36). However, presence of antigen doesn't necessarily signify the presence of a viable, well-established cysticercus infection. It could be indicative for short term partial establishment, and perhaps a ‘transient’ antigen presence should be considered (37). A Zambian study demonstrated the rather short-term presence of circulating antigen in 31 participants.Whether this observation is due to a partial establishment of infection (immature cysticerci), or the establishment of cysticerci, followed by a quick degeneration (self cure?) could not be confirmed. The Ts Ag results in that study were provided as qualitative data.It may be that antigen levels (ratio) can add complementary information. Indeed, Mwape et al (37) observed that individuals who became seronegative were those that displayed low levels of circulating antigen. Therefore, serological results from field studies should be looked at critically. Individuals with positive test results shouldn't be by definition considered as infected with*T. solium*, but the possibility of a transient antigen response has to be taken into account.

The benefit of treatingchildren positive for Ts antigen is still debated, and the medical interventions should be carried out in neurology Units. However, in Madagascar neurologists are available only in Antananarivo and Mahajunga, and not in the cities under study. Treating a positive case without a proper medical examination may impair and even endanger the child’s life and by destroying viable cysts, it may enhance the brain seizures with a high risk of morbidity and even mortality (9).

Limitations

This is a cross-sectional study in schoolchildren from seven sites in the four different biotopes of Madagascar. The samples were collected 13 years ago. It may be suspected that risk factors have changed. However, according to Word Bank data (accessed 12/21/2020): GDP per capita (current USD) decreased from 536 to 523 USD. Assuming that the risk factors are associated with poverty and haven’t significantly changed, the obtained data may be extrapolated to the current years.

The main limitations of our study are the lack of clinical data and risk factors associated with cysticercosis.

- The blood samples were collected in the framework of a malaria study. Therefore, factors recorded were not specific to cysticercosis. Further and updated studies should rely on prospective studies focusing on social, behavioural, sanitary, economic determinantsand the Taeniasis/Cysticercosis risk factors that may play a role in the spread of the disease and explain the differences between sites. Implementing specifically cysticercosis driven prospective studies will enable to show the dynamic nature of *T. solium* infections in the different biotopes and adapt accordingly human and animal health policies.
- Clinical features have to be taken into account and a possible follow-up should be offered to patients featuring a probable/possible diagnosis of NCC (38). The presence of a positive Ag Ts is not sufficient to diagnose NCC since its positivity does not indicate a neurological localization and an important number of infections probably never fully establish, leading to possibly ‘transient’ antigen presence (37). Furthermore, in humans, it is described that cysticerci may stay viable during years according to their localization (3). Many factors, among which, the size of the (re) infection, the immune status of the host, age and sex play a determining role in the (non-) establishment of infection (39).

Clinical features including epilepsy are critical to define a diagnosis of NCC added to imaging data, laboratory findings and epidemiology patterns (38). Many reports described cysticercosis-associated epilepsy in Madagascar (40): pediatricians of the military hospital of Antananarivo reported that epilepsy was present in over 80% of the cases of NCC. An interesting finding is that the seroprevalence (Ts Ab) found among suspected cases was between 25 and 48% (ELISA+/-EITB) (40).However, those studies were completed between 1991 to 1993 and did not use Ts Ag as the diagnostic tool. Therefore, unpublished data communicated in 2012, displayed a Ts Ag positivity of 73% among patients having clinical and imaging features attributable to NCC (41). Clinical symptoms and biological results are, however, not sufficient for a diagnosis of NCC (38). CT-scan/MRI are essential for this purpose: they are the sole way to assess the number, the neurological localisation and the status (viable/degenerated/calcified) of cysticerci. Unfortunately, these imaging tools are obviously not applicable and available for field studies.
